# Desorption efficiency and holding capacity of acid-treated filters for nicotine sampling in vape shops

**DOI:** 10.1093/annweh/wxae080

**Published:** 2024-10-25

**Authors:** Toluwanimi M Oni, Sanjeewa Gamagedara, Evan L Floyd

**Affiliations:** Department of Occupational and Environmental Health, Hudson College of Public Health, University of Oklahoma Health Sciences Center, 801 NE 13th Street, Oklahoma City, OK 73104, United States; Department of Chemistry, University of Central Oklahoma, 100 N. University Dr., Edmond, OK 73034, United States; Department of Occupational and Environmental Health, Hudson College of Public Health, University of Oklahoma Health Sciences Center, 801 NE 13th Street, Oklahoma City, OK 73104, United States

**Keywords:** electronic cigarette, exposure assessment, nicotine, vape shop

## Abstract

Efficient sampling materials are essential for assessing nicotine levels in vape shops and other settings where nicotine exposures may exist. Two different treatments of Whatman glass fiber type A (GF/A) filters (sodium bisulfate treated and citric acid treated) were evaluated for nicotine capture, desorption efficiency, and holding capacity using Gas Chromatography–Mass Spectrometry (GC–MS). The Filters were treated with 0.8 mL of 0.1 M sodium bisulfate or citric acid solution and oven-dried (80 °C) for 30 min. Nicotine was desorbed off the filters using a modified analytical method. The average nicotine desorption efficiency for sodium bisulfate-treated GF/A filters (98.4%) was significantly higher than that of citric acid-treated GF/A filters (60.9%) over a range of 1–100 µg nicotine. Sodium bisulfate-treated and citric acid-treated GF/A filters experienced a 10% nicotine breakthrough after being dosed with about 550 and 2,750 µg of nicotine, respectively compared to 75 µg for untreated GF/A filters. Citric acid-treated GF/A filters had a much greater nicotine-holding capacity, but nicotine desorption from citric acid-treated GF/A filters was below the recommended criteria. Therefore, we recommend that sodium bisulfate-treated GF/A filters are employed for sample of nicotine with the GC–MS method.

**What’s Important About This Paper?**

This study evaluates the desorption efficiency and storage or holding capacity of 2 acid-treated filter types for sampling and analysis of airborne nicotine in electronic cigarette aerosol. This work is important because vape shops have emerged as sources of occupational exposure to nicotine, even as bans on smoking and vaping in workplaces have increased in recent years.

## Introduction

The vape shop environment could possibly expose workers to elevated nicotine levels compared to the average electronic cigarette (EC) user (Son et al. 2020; Li et al. 2021) and this is due to the prevalence of in-store vaping (Zwack et al. 2017; Son et al. 2020; Zhang et al. 2020; Li et al. 2021). Unlike most retail outlets that do not have significant exposure to chemicals and aerosols, the typical vape shop environment’s visible haze, and a distinct odor provide indication of potential exposures to vapors (Son et al. 2020; Attfield et al. 2022).

To assess nicotine levels present in vape shops and other high tobacco-use settings, efficient sampling materials need to be deployed treated filters have been widely used for nicotine sampling (Gorini et al. 2004; Fu et al. 2013; Arechavala et al. 2018; Díez-Izquierdo et al. 2018; Feliu et al. 2020) apart from the National Institute for Occupational Safety and Health (NIOSH) method for nicotine sampling (NMAM 2551) (NIOSH, 1998) involving XAD-4 sorbent tubes. Two commonly used treated filters for nicotine sampling are sodium bisulfate-treated filters (Hammond et al. 1987; Ogden and Maiolo 1992; Gorini et al. 2004; Fu et al. 2013; Arechavala et al. 2018; Díez-Izquierdo et al. 2018; Feliu et al. 2020) and citric acid-treated filters (Koutrakis et al. 1989; Caka et al. 1990; Lewis et al. 1994).

A major disadvantage of the NMAM 2551 method was observed by Ogunwale et al. (2017) showing nicotine collection by XAD-4 sorbent tubes were consistently lower than an impinger method due to incomplete capture of nicotine during sampling and incomplete desorption during analysis. Additionally, the NMAM 2551 recommends a filter be used in series when nicotine is being sampled amidst particulates, but no filter when sampling environmental tobacco smoke (ETS). Though conceptually analogous to ETS, environmental EC aerosol is not smoke and could be interpreted at a fog or haze. Using only a treated filter to capture nicotine from both particle and vapor phases presents a simplification over the XAD-4 sorbent approach. Lastly and possibly most importantly, the measurement of a liquid particulate matter (PM) is challenging due to evaporation. Treated filters can be combined with real-time particulate monitors for characterization of EC particle content and total nicotine capture. This study was therefore aimed at evaluating and comparing the desorption efficiencies and holding capacities of sodium bisulfate-treated and citric acid-treated filters for nicotine sampling in vape shops.

## Materials and methods

Whatman GF/A glass microfiber filters (47 mm, ^©^Cytiva) were treated with 0.08 mmol of sodium bisulfate or citric acid, which kept parity with Hammond et al.’s study (1987). Filters were treated with 0.8 mL of 0.1 M sodium bisulfate or citric acid solution and oven-dried (80 °C) for 30 min. Sodium bisulfate-treated and citric acid-treated filters were evaluated for their nicotine desorption efficiency for 5 different nicotine masses (1, 5, 10, 50, and 100 µg) (See Table 1).

**Table 1.** Nicotine recovery (%) from sodium bisulfate-treated and citric acid-treated filters.

Nicotine(μg)	Sodium bisulfate		Citric acid	
	Nicotine recovery (%)	RSD (%)	Nicotine recovery (%)	RSD (%)
1	102	5.6	<RL^*^	-
5	95.9	4.2	42.8	33
10	96.7	9.5	70.5	8.0
50	100	1.5	90.9	4.8
100	96.7	8.8	100	8.0

^*^RL = reportable limit.

## Procedures

### Nicotine capture and desorption efficiency

The treated filters were evaluated for their nicotine desorption efficiency at 5 different nicotine masses (1, 5, 10, 50, and 100 µg) with 5 replicates at each mass. Analysis was performed using the modified gas chromatography–mass spectrometry (GC–MS) analytical method. Desorption efficiency of recovery (%) was computed as:


$$\begin{array}{l} Recovery\;\left(\%\right)=\frac{Predicted\;mass}{Spiked\;mass}\times 100. \end{array}$$ (1)

### Holding capacity of acid-treated filters

**Fig. 1.**
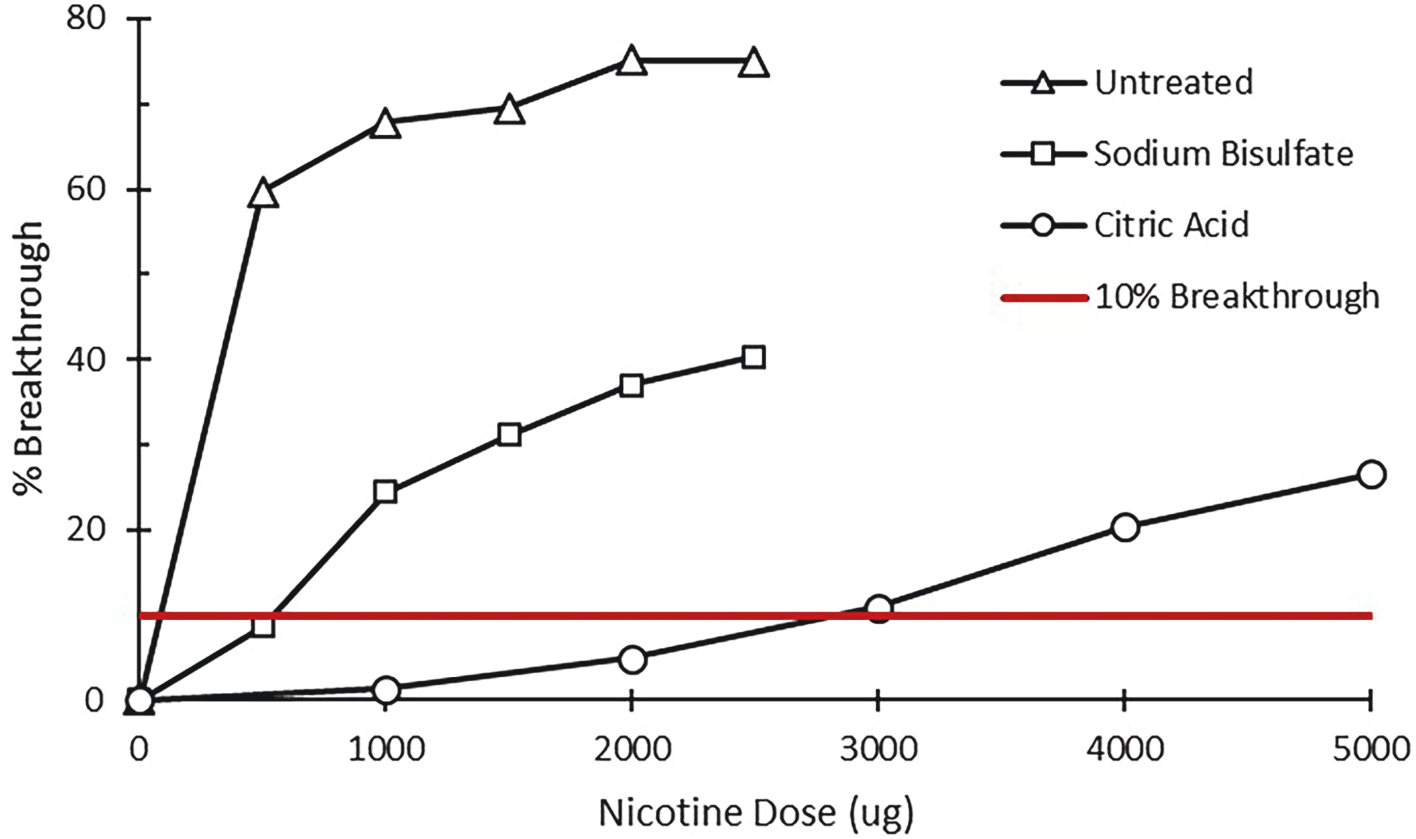
Nicotine breakthrough (%) for untreated, sodium bisulfate, and citric acid-treated filters with 10% breakthrough shown as solid (red) horizontal line.

The amount of nicotine that can be captured by treated filters was qualitatively assessed by performing a simple breakthrough capacity experiment (*n* =1 for each treatment type). Two treated filters were placed in series in open-face sampling cassettes (SKC Ltd., Eighty Four, PA) and sampled at 2 L/min. The upstream filter was the treatment being tested for breakthrough (sodium bisulfate or citric acid), and the downstream filter served as the analytical filter to quantify breakthrough. The downstream filter was always a sodium bisulfate-treated filter. Spikes of nicotine vapor were administered via a heated glass tube at 175 °C. Nicotine dissolved in isopropanol was spiked in small increments (500 or 1,000 µg) into the heated tube and drawn as a vapor onto the filter cassette for 1 min. After each spike, the downstream filter was replaced with a fresh sodium bisulfate-treated filter and later analyzed (Fig. S1). To create a breakthrough curve, the cumulative nicotine dosed on the upstream filter was plotted versus the cumulative percent breakthrough on the downstream filters. Using these breakthrough curves (Fig. 1), the 10% breakthrough capacity was estimated to determine which treatment had a greater holding capacity. The breakthrough capacity of an untreated filter was assessed in like manner for comparison.

### Gas chromatography–mass spectrometry

A new GC–MS method was developed by adapting a gas chromatography–nitrogen–phosphorus detector (GC–NPD) analysis method originally described by Hammond et al. (1987) involving the extraction of nicotine from diesel particulates into ammoniated heptane. Important adaptations to the Hammond et al. (1987) method include using 10 µL of sodium hydroxide (instead of 2 mL), extracting nicotine into dichloromethane instead of ammoniated heptane, and use of a mass spectrometer (MS) instead of an NPD.

The GC–MS instrument setup used in this study was an Agilent 6890 GC, 5973-N Mass Selective Detector, split/splitless injector operated in splitless mode, DB-5 capillary column (30 m × 0.25 mm × 0.5 µm), 1.5 mL/min column flow rate, 1 µL injection volume, 250 °C inlet temperature, 120 °C oven initial temperature, 0.5 min oven hold, 15 °C/min oven ramp to 150 °C, 50 °C/min ramp to 300 °C, followed by 2.5 min hold. The MS was operated in scan mode. Ions of interest were 162, 161, 133, and 84 *m/z*, quantification based on 84 *m/z*. Total run time was 8 minutes, and retention time was approximately 5.0 minutes. Quinoline was used as an internal standard at 50 μg/mL. We suggest using a 10-fold lower level of internal standard.

## Results

### Nicotine recovery from treated filters

Nicotine recoveries (%) and relative standard deviation(RSD) from the treated filters are presented in Table 1. The adapted GC-MS technique was used to analyze and compare nicotine capture and desorption of sodium bisulfate-treated and citric acid-treated filters. Sodium bisulfate-treated filters had recoveries ranging from 95.9% to 102% for the 5 nicotine masses evaluated with an average recovery of 98.4%. Recoveries from citric acid-treated filters ranged from <RL—100% with an average of 60.9%.

### Holding capacity of untreated and acid-treated filters

The nicotine-holding capacity of treated and untreated GF/A filters are illustrated in Fig. 1. For the untreated filter, a benchmark 10% nicotine breakthrough was estimated as 75 µg of nicotine vapor. For sodium bisulfate-treated and citric acid-treated filters, the 10% nicotine breakthrough capacity was estimated as 550 and 2750 µg nicotine vapor, respectively. Both treated filters had substantially greater holding capacity than the untreated filter. Expectedly, the citric acid-treated filter had the highest nicotine-holding capacity of the 3 filter types.

## Discussion

A sampling and analytical method used to quantify exposure samples should span 0.1–2 times the mass collected for exposures at the occupational expsoure limit (OEL) and should have a desorption efficiency of ≥ 75%, but greater than 90% is preferred (Eide 2010). Filters treated with sodium bisulfate had consistently high desorption efficiency (98.4% mean) at all nicotine masses (1–100 µg). The holding capacity was close to the upper analytical range and could easily be increased by loading more sodium bisulfate on the filter during treatment. Citric acid, however, had desorption efficiency below the recommended criteria at 1, 5, and 10 µg, but approximately 5 times greater holding capacity than sodium bisulfate. Use of citric acid as a chemisorbent for nicotine should be viable for samples with >100 µg nicotine but would have approximately 100 times poorer sensitivity than sodium bisulfate-treated filters and would not have adequate sensitivity for a typical air sample collected at the OEL using an optical particle counter with a flow rate of 1.2 L/min (29–576 µg nicotine analytical range needed).

For high flow rate sampling or high exposure situations, either sodium bisulfate or citric acid-treated filters could be used for nicotine collection but citric acid may be preferred due to their 5-fold greater holding capacity at equal molar treatment as sodium bisulfate. To use sodium bisulfate under these conditions the GF/A filters will need to be treated with more sodium bisulfate. Citric acid’s organic nature and triprotic structure are responsible for the 5 times greater holding capacity even though the acid normality was only 3 times greater than sodium bisulfate. These attributes may also be responsible for the poor recovery below 50 µg.

## Conclusion

Nicotine was more efficiently desorbed from sodium bisulfate-treated filters compared to citric acid-treated filters. Thus, sodium bisulfate-treated GF/A filters are the preferred option for occupational and environmental sampling for nicotine in settings such as vape shops. However, citric acid’s strong affinity for nicotine and high holding capacity may be better suited for environmental nicotine sequestration.

## Supplementary material

Supplementary material is available at *Annals of Work Exposures and Health* online.

wxae080_suppl_Supplementary_Figures_S1_Tables_S1-S2

## Data Availability

The data collected during this study is presented in their entirety in the Tables S1 and S2.
