# Supplementary material for: Desorption efficiency and holding capacity of acid-treated filters for nicotine sampling in vape shops
Source: Ann Work Expo Health. 2024 Oct 25;69(1):104–8. doi: 10.1093/annweh/wxae080 (PMC11706789; doi:10.1093/annweh/wxae080)
Supplement: wxae080_suppl_Supplementary_Figures_S1_Tables_S1-S2 [file wxae080_suppl_supplementary_figures_s1_tables_s1-s2.pdf]

DESORPTION EFFICIENCY AND HOLDING CAPACITY OF ACID-TREATED FILTERS FOR NICOTINE SAMPLING IN VAPE SHOPS

Toluwanimi M. Oni MPH[1] Sanjeewa Gamagedara PhD[2] and Evan L. Floyd PhD[1]

[1]Department of Occupational and Environmental Health, Hudson College of Public Health, University of Oklahoma Health Sciences Center, Oklahoma City, OK 73104, µSA

[2]Department of Chemistry, University of Central Oklahoma, Edmond, OK 73034, µSA

Corresponding Author:

Evan L. Floyd PhD

Department of Occupational and Environmental Health,

Hudson College of Public Health,

University of Oklahoma Health Sciences Center,

Oklahoma City, OK 73104, USA

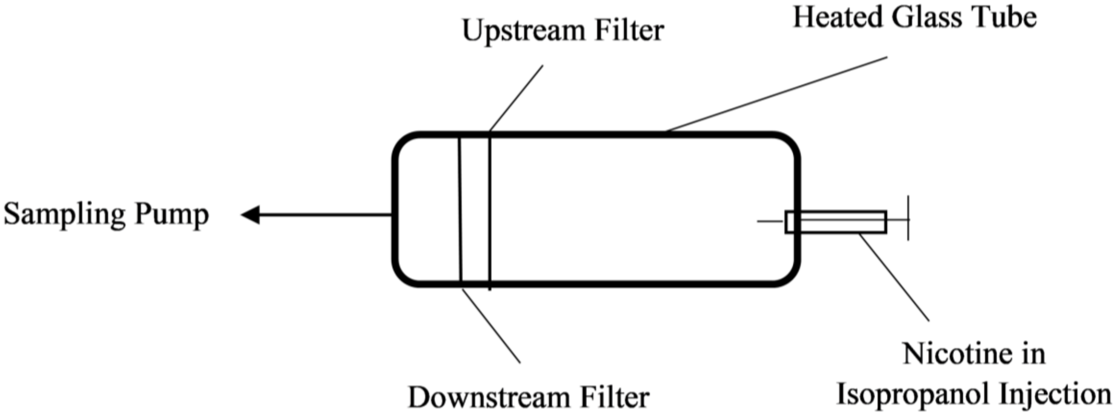

Figure S1. Experimental Setup for Nicotine Holding Capacity of Treated and Untreated Filters

Table S1. Nicotine Recovery from Sodium Bisulfate-Treated and Citric Acid-Treated Filters

**Acid-Mass**	**Nicotine (µg)**	**%Yield**	**Acid-Mass**	**Nicotine (µg)**	**%Yield**
SB 1µg -1	1.06	106	CA 1µg -1	<RL	<RL
SB 1µg -2	1.09	109	CA 1µg -2	<RL	<RL
SB 1µg -3	1.00	100	CA 1µg -3	<RL	<RL
SB 1µg -4	0.95	95	CA 1µg -4	<RL	<RL
SB 1µg -5	1.01	101	CA 1µg -5	<RL	<RL
SB 5µg -1	4.62	92	CA 5µg -1	3.00	60
SB 5µg -2	5.03	101	CA 5µg -2	1.86	37
SB 5µg -3	4.76	95	CA 5µg -3	1.37	27
SB 5µg -4	4.98	100	CA 5µg -4	2.32	46
SB 5µg -5	4.59	92	CA 5µg -5	NA	NA
SB 10µg -1	9.57	96	CA 10µg -1	6.59	66
SB 10µg -2	9.87	99	CA 10µg -2	6.40	64
SB 10µg -3	8.76	88	CA 10µg -3	7.64	76
SB 10µg -4	9.04	90	CA 10µg -4	7.59	76
SB 10µg -5	11.11	111	CA 10µg -5	7.05	70
SB 50µg -1	49.82	100	CA 50µg -1	42.48	85
SB 50µg -2	50.87	102	CA 50µg -2	44.68	89
SB 50µg -3	49.61	99	CA 50µg -3	47.20	94
SB 50µg -4	51.24	102	CA 50µg -4	44.99	90
SB 50µg -5	49.61	99	CA 50µg -5	47.90	96
SB 100µg -1	95.36	95	CA 100µg -1	109.25	109
SB 100µg -2	96.97	97	CA 100µg -2	103.65	104
SB 100µg -3	88.09	88	CA 100µg -3	88.02	88
SB 100µg -4	92.45	92	CA 100µg -4	103.01	103
SB 100µg -5	110.67	111	CA 100µg -5	97.42	97

NA – Data unavailable due to poor injection during sample analysis

Table S2. Nicotine Breakthrough for Citric Acid, Sodium Bisulfate Treated and Untreated Filters

Citric Acid

Nicotine Dose (µg)	Nicotine Breakthrough Mass (µg)	Cumulative Nicotine Breakthrough Mass (µg)	Nicotine Breakthrough (%)
0	0	0	0
1000	13.87	13.87	1
2000	86.01	99.88	5
3000	226.56	326.44	11
4000	487.84	814.28	20
5000	512.73	1327.01	27

Sodium Bisulfate

Nicotine Dose (µg)	Nicotine Breakthrough Mass (µg)	Cumulative Nicotine Breakthrough Mass (µg)	Nicotine Breakthrough (%)
0	0	0	0
500	43.13	43.13	9
1000	200.79	243.92	24
1500	222.5	466.42	31
2000	273.94	740.36	37
2500	268.63	1008.99	40

Untreated

Nicotine Dose (µg)	Nicotine Breakthrough Mass (µg)	Cumulative Nicotine Breakthrough Mass (µg)	Nicotine Breakthrough (%)
0	0	0	0
500	298.66	298.66	60
1000	379.45	678.11	68
1500	363.99	1042.1	69
2000	459.88	1501.98	75
2500	372.51	1874.49	75
